# Genome-Wide Identification and Expression Profile of *OSCA* Gene Family Members in *Triticum aestivum* L.

**DOI:** 10.3390/ijms23010469

**Published:** 2021-12-31

**Authors:** Kai Tong, Xinyang Wu, Long He, Shiyou Qiu, Shuang Liu, Linna Cai, Shaofei Rao, Jianping Chen

**Affiliations:** 1 State Key Laboratory for Managing Biotic and Chemical Threats to the Quality and Safety of Agro-Products, Key Laboratory of Biotechnology in Plant Protection of Ministry of Agriculture and Zhejiang Province, Institute of Plant Virology, Ningbo University, Ningbo 315211, China; 2011074042@nbu.edu.cn (K.T.); qiushiyoudyzx@126.com (S.Q.); liushuang4060@126.com (S.L.); cln7471lcy@163.com (L.C.); 2 College of Life Science, China Jiliang University, Hangzhou 310058, China; xy_wu@zju.edu.cn; 3 Ministry of Agriculture Key Lab of Molecular Biology of Crop Pathogens and Insects, Institute of Biotechnology, College of Agriculture & Biotechnology, Zhejiang University, Hangzhou 310058, China; hnndhelong2@163.com

**Keywords:** abiotic stress, *reduced hyperosmolality-gated calcium-permeable channels*, wheat, whole genome expression analysis

## Abstract

Hyperosmolality and various other stimuli can trigger an increase in cytoplasmic-free calcium concentration ([Ca^2+^]_cyt_). Members of the *Arabidopsis thaliana* (L.) *reduced hyperosmolality-gated calcium-permeable channels* (*OSCA*) gene family are reported to be involved in sensing extracellular changes to trigger hyperosmolality-induced [Ca^2+^]_cyt_ increases and controlling stomatal closure during immune signaling. Wheat (*Triticum aestivum* L.) is a very important food crop, but there are few studies of its *OSCA* gene family members. In this study, 42 *OSCA* members were identified in the wheat genome, and phylogenetic analysis can divide them into four clades. The members of each clade have similar gene structures, conserved motifs, and domains. *TaOSCA* genes were predicted to be regulated by cis-acting elements such as STRE, MBS, DRE1, ABRE, etc. Quantitative PCR results showed that they have different expression patterns in different tissues. The expression profiles of 15 selected *TaOSCAs* were examined after PEG (polyethylene glycol), NaCl, and ABA (abscisic acid) treatment. All 15 *TaOSCA* members responded to PEG treatment, while *TaOSCA12*/-*39* responded simultaneously to PEG and ABA. This study informs research into the biological function and evolution of *TaOSCA* and lays the foundation for the breeding and genetic improvement of wheat.

## 1. Introduction

Under natural conditions, plants encounter a variety of biotic and abiotic stresses [1,2]. Plants resist these stresses by sensing and transmitting signals in a variety of ways that regulate responses and gene expression, thereby producing appropriate physiological and morphological changes [3,4,5]. In the process of signal perception, calcium ions (Ca^2+^) are important second messengers when plants respond to stress [6]. Under osmotic stress, plants induce a rapid increase in intracellular free Ca^2+^ concentration ([Ca^2+^]_cyt_), thereby inducing the expression of many stress-related genes and regulating the tolerance of plants to osmotic stress [4,7]. Blocking hyperosmolality-induced [Ca^2+^]_cyt_ increase (OICI) interferes with gene expression induced by drought, indicating that the precise regulation of OICI is essential for the activation of many signal transduction pathways triggered by external stimuli [7]. The increase in intracellular Ca^2+^ concentration is mainly regulated by calcium transport systems such as calcium channels and calcium pumps [8]. Previous studies have shown that osmotic/mechanical stimulus-gated calcium permeable channels play the role of osmosensors in bacteria and animals [9,10], which indicates that there may be specific calcium-permeable channels in plants that function as osmosensors [11,12].

Some protein families, including glutamate receptor-like proteins (GLR), cyclic nucleotide gated channels (CNGCs), and annexins, are involved in calcium flux in plant cells [13]. However, none of them can directly respond to pressure signals [14]. In 2014, Yuan et al. used a genetic screening strategy based on calcium imaging to isolate *Arabidopsis thaliana* (L.) mutants with a low intracellular free calcium concentration under high osmotic stress and characterized the gene *reduced hyperosmolality-induced [Ca^2+^]_cyt_ increase 1* (*osca1.1*) as the previously unknown hyperosmolality-gated calcium-permeable channel [15]. An *osca1.1* mutant had impaired osmotic calcium signal transmission in guard cells and root cells and impaired regulation of water transpiration and root growth in response to osmotic stress, indicating that *osca1.1* may be an osmosensor in *A. thaliana* [15]. OSCA1.1 belongs to a 15-member gene family in *A. thaliana*, and homologous genes are found in other plant species and eukaryotes [16,17,18]. Studies on functional domains indicate that the OSCA gene family encodes a calcium-dependent channel domain (DUF221) [14]. DUF221 belongs to the anoctamin-like family and is homologous to the domain in the anoctamin/TMEM16 channel, which is a Calcium-activated Chloride Channel (CaCC) component and transmembrane channel-like protein (TMC) [19]. DUF221 proteins are a family of osmosensitive calcium-permeable cation channels, which are conserved in eukaryotes [20]. By heterologous expression of *A. thaliana* genes in Chinese Hamster Ovary (CHO) cells loaded with calcium-sensitive dye Fura-2, Hou et al. screened OSCA1.2 as an ion channel that can be activated by hyperosmotic shock. Detailed analysis showed that OSCA1.2 has good permeability to cations such as Ca^2+^, K^+^, and Na^+^ [14]. OSCA3.1 was previously reported to be an early response protein to dehydration stress in *A. thaliana* [21]. However, Yuan et al. reported that OSCA3.1 knockout mutants displayed normal OICIs, suggesting that the function of OSCA3.1 may be different from that of OSCA1.1 [15].

The perception of biotic and abiotic stresses often leads to the closure of plant stomata [1,22], and the rapid influx of calcium through the plasma membrane plays an important role in this response [23,24]. However, the identity of the calcium channel was unclear until Thor et al. recently reported that the *A. thaliana* Ca^2+^-permeable channel OSCA1.3 controls stomatal closure in the process of immune signal transduction [25]. OSCA1.3 is permeable to Ca^2+^ and can be rapidly phosphorylated by the receptor-like cytoplasmic kinase protein BIK1 when sensing Pathogen-Associated Molecular Patterns (PAMPs). Genetic and electrophysiological data reveal that in the process of immune signal transduction, BIK1-mediated N-terminal phosphorylation of OSCA1.3 increases the activity of this channel [25]. However, OSCA1.3 does not regulate stomatal closure induced by abscisic acid (ABA, a plant hormone related to abiotic stress) [25]. In summary, the members of the OSCA family are both conserved and differentiated in function.

OSCA family members thus play a vital role in plant resistance to high osmotic stress and other stimuli. The *OSCA* gene family has been systematically identified and analyzed in *A. thaliana* [14,15], rice [16], maize [18], and *Vigna radiate* L. [17] but not so far in wheat (*Triticum aestivum* L.), globally one of the most widely grown crops [26]. To explore whether the wheat *OSCA* gene family has a function in the process of regulating abiotic stress response, we conducted a genome-wide identification of *T. aestivum* OSCA family members and analyzed their phylogenetic relationships and expression profiles under different tissues and different abiotic stresses in this study. These results lay the foundation for studying the function of the wheat *OSCA* genes and increase our understanding of the role of plant *OSCA* genes in general.

## 2. Results

### 2.1. Genome-Wide Identification and Naming of TaOSCA Members

We identified the possible OSCA members in *Triticum aestivum* based on the criterion that the OSCA gene contains a conserved DUF221 functional domain (pfam accession number: 02714). The amino acid sequences of 15 identified *A. thaliana* OSCA members were downloaded from TAIR, and the *T. aestivum* genome sequence and gene structure annotation files were downloaded from the Ensemble Plants database (http://plants.ensembl.org/Triticum_aestivum/Info/Index, accessed on 29 December 2021). A total of 42 TaOSCAs were identified after two rounds of BLASTP. These members are named TaOSCA1 to TaOSCA42 according to their position on the chromosomes (Table 1 and Supplementary Table S1). The proteins range in size from 469 to 804 amino acids (aa), with most having about 700 aa. Their molecular weights are 53.7–93.4 kDa, and the isoelectric points are in the range of 6.59–9.77.

### 2.2. Phylogenetic Analysis

**Figure 1:**
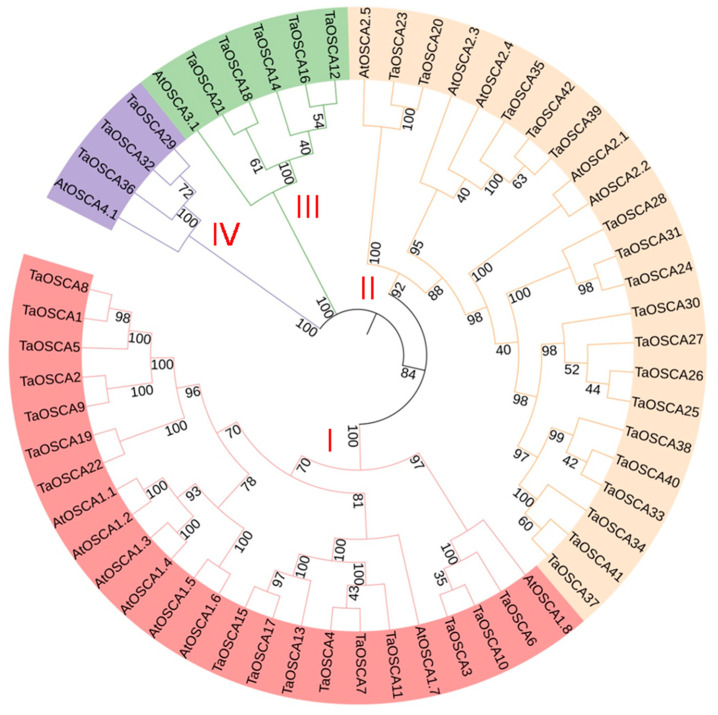
Phylogenetic tree of the OSCA proteins of *Triticum aestivum* and *A. thaliana***.** The phylogenetic tree was constructed using OSCA amino acid sequences by the neighbor-joining method in MEGA X with 1000 bootstrap replicates. The phylogenetic tree is divided into four groups, which are shown in different colors, and identified by red Roman numerals.

In order to better understand the evolutionary relationship of *OSCA* family genes in *A. thaliana* and *T. aestivum*, the amino acid sequences of 15 *A. thaliana* and 42 *T. aestivum* members were used to construct a phylogenetic tree. The genes clearly divide into four clades (I to IV; Figure 1). There are 16 TaOSCA members in clade I (TaOSCA1, -2, -5, -8, -9, -19, -22, -4, -7, -11, -13, -15, -17, -3, -6, -10) and seven of *A. thaliana* (AtOSCA1.1-AtOSCA1.7). Clade II contains 18 wheat (TaOSCA20, -23, -39, -42, -35, -24, -31, -28, -25, -26, -27, -30, -33, -40, -38, -34, -37, -41) and four *A. thaliana* (AtOSCA2.1-AtOSCA 2.4) members. There are five wheat members in clade III (TaOSCA18, -21, -14, -12, -16), together with AtOSCA3.1, and three wheat members in clade IV (TaOSCA36, -29, -32) with AtOSCA4.1 (Figure 1).

### 2.3. Analysis of TaOSCA Conserved Motifs, Gene Structure, and Domains

**Figure 2:**
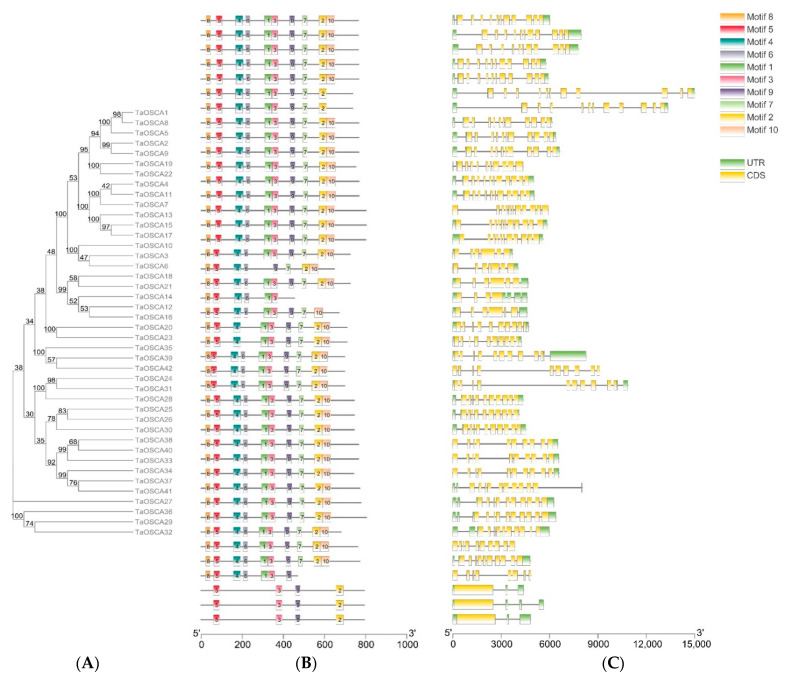
Analysis of conserved motifs and gene structure of *T. aestivum OSCA* genes. (**A**) Phylogenetic tree constructed using the TaOSCA protein sequences. (**B**) Ten types of conserved motifs predicted in the TaOSCA protein sequences. The different motifs are shown in different color boxes. The sequence information for each motif is provided in Supplementary Table S2. (**C**) The gene structure of *TaOSCA* members. Untranslated regions, exons, and introns are shown as green boxes, yellow boxes, and horizontal lines, respectively.

To further understand the evolution of TaOSCA members, we compared the conserved motifs, exon-intron composition, and functional domains of the 42 TaOSCA members. An online MEME analysis predicted a total of 10 conserved motifs (Supplementary Table S2). The vast majority of members contain all 10 predicted motifs, but the three members of clade IV contain only four motifs (Figure 2A,B). TaOSCA12 contains six motifs, TaOSCA27 contains seven motifs, TaOSCA21 contains eight motifs, and TaOSCA19/-22/-16 contain nine motifs (Figure 2A,B). The *TaOSCA* gene structure is relatively conserved with most having about 10 introns, while the three members of the fourth clade have only 2–3 introns (Figure 2C).

**Figure 3:**
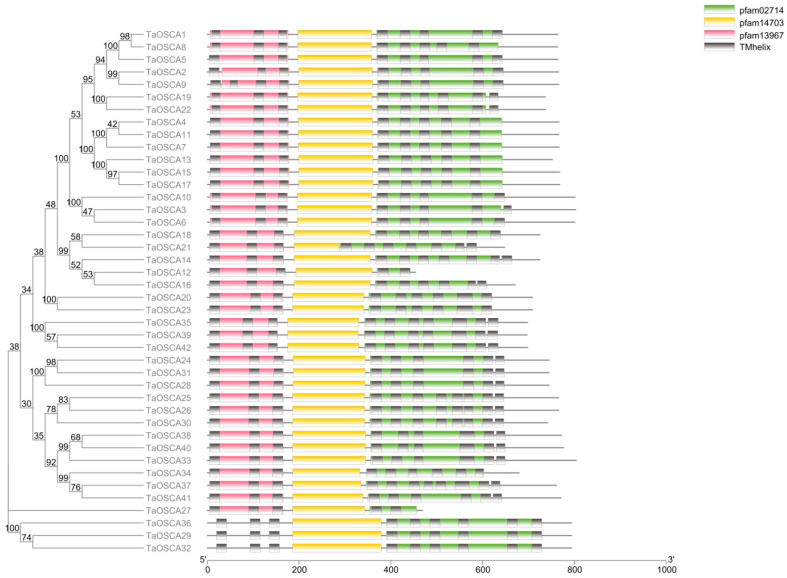
Analysis of conserved domains in TaOSCA proteins. Pink, yellow, green, and gray boxes represent the pfam13967, pfam14703, pfam02714, and transmembrane helix domain, respectively.

All 42 members contain the conserved pfam02714 and pfam14703 functional domains. Except for the three members of the fourth clade, the other members also contain a pfam13967 functional domain (Figure 3). Pfam02714 is predicted to be the transmembrane region of the osmosensitive calcium-permeable cation channel [14,27,28]. The functional domain of pfam13967 is predicted to be the first three transmembrane regions of transmembrane proteins, which is related to vesicle transport [27]. pfam14703 is predicted to be the cytoplasmic region of an integral membrane protein. This functional domain usually appears before pfam02714 and after pfam13967 [28]. In addition, most TaOSCA genes contain 8–10 transmembrane regions. The exceptions are TaOSCA25/-26/-30/-37, which have 11 transmembrane regions, and TaOSCA12/-27, which have only five (Figure 3).

### 2.4. Prediction of Cis-Acting Elements of the TaOSCA Promoter

**Figure 4:**
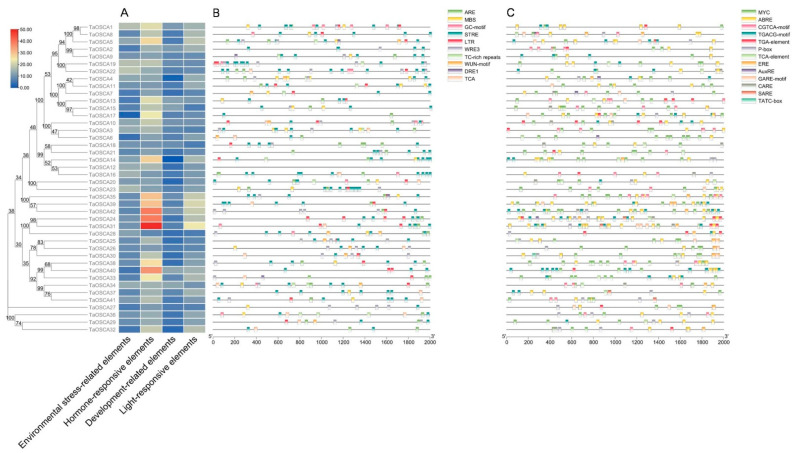
Prediction of cis-acting elements in the *TaOSCA* promoter regions. (**A**) Schematic representation of the numbers of four types of cis-acting elements predicted in the promoter region of each *TaOSCA* member. (**B**,**C**) The type, quantity, and position of environmental stress-related elements (**B**) and hormone-response elements (**C**) in the promoter region.

The cis-acting elements in the *TaOSCA* promoter region were then analyzed, and a total of 107 types of 6542 cis-acting elements were predicted (Supplementary Table S3). These cis-acting elements are related to environmental stress, hormonal response, development, and light response (Figure 4A). A total of 385 elements related to environmental stress are predicted in 10 categories, among which the number of STRE, ARE, and WUN-motif elements is relatively large (Figure 4B). A total of 806 hormone-related components are predicted in 13 categories, of which ABRE, MYC, and CGTCA-motif components are relatively large, mainly related to ABA and JA (Figure 4C). This indicates that *TaOSCA* family genes may be involved in a variety of stress and plant hormone response processes and can effectively promote plant growth and stress resistance.

### 2.5. Expression Patterns of TaOSCA Genes in Different Tissues

**Figure 5:**
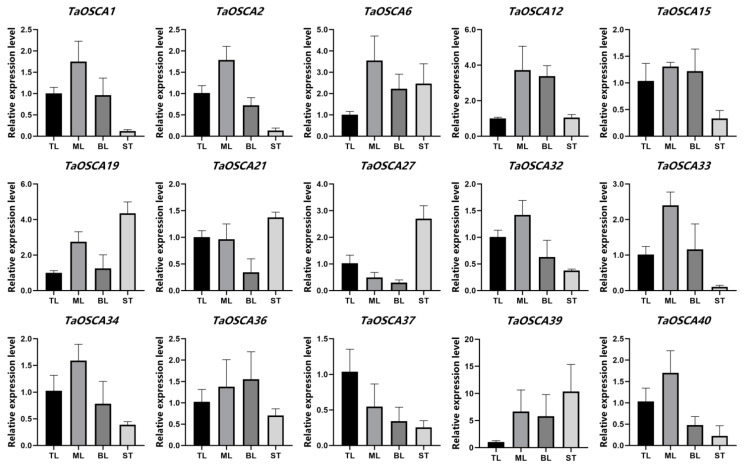
Expression levels of *TaOSCAs* in different tissues. The mean expression values were calculated from three independent biological replicates relative to that in top leaves. TL: top leaf; MF: middle leaf; BF: bottom leaf; ST: stem.

To study the expression pattern of *TaOSCA* genes, the expression levels of 15 *TaOSCA* members representing the different clades were analyzed in five different tissues (root, stem, bottom leaf, middle leaf, top leaf). Gene expression in the roots was always very low or undetectable. *TaOSCA37* was expressed most highly in the top leaf, whereas *TaOSCA6*/-*39* were expressed least in this leaf (Figure 5). *TaOSCA1/-2/-6/-12/-32/-33/-34/-39/-40* were expressed most highly in the middle leaf (Figure 5). *TaOSCA39* was expressed more highly in the bottom leaf than the top leaf, *TaOSCA21/-27/-40* had lower expression levels in the bottom leaf, and other members were expressed at similar amounts in the bottom and top leaves (Figure 5). In addition, *TaOSCA6/-19/-27/-39* were expressed most highly in the stem (Figure 5).

### 2.6. Expression Profiles of TaOSCA Genes in Response to Abiotic Stress Treatments

**Figure 6:**
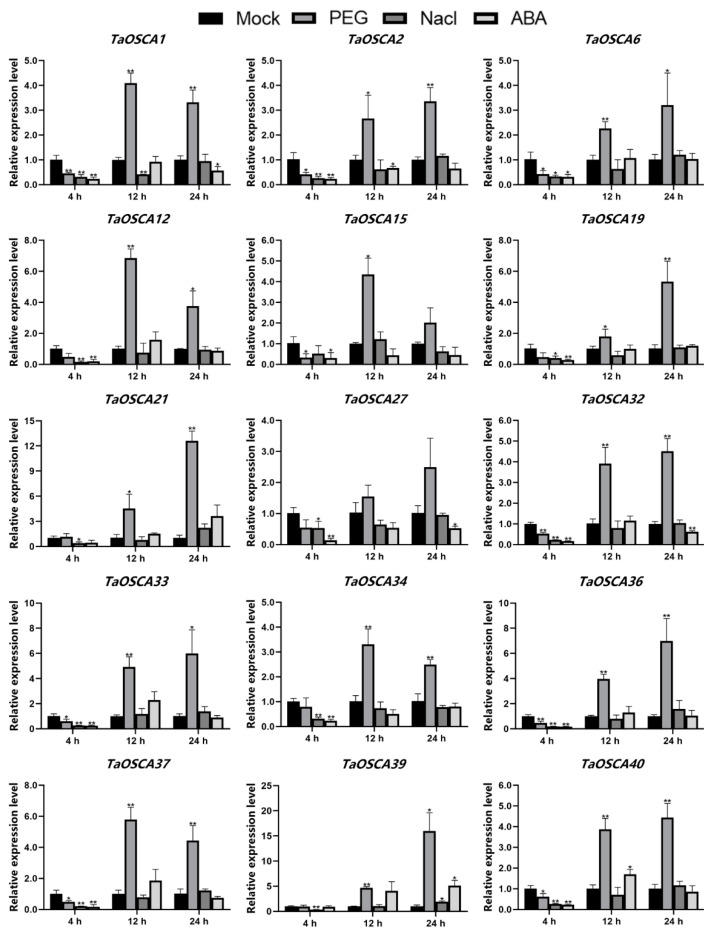
Expression analysis of *TaOSCAs* under PEG, NaCl, and ABA treatment. Gene expression was normalized to control unstressed expression level, which was assigned a value of 1. Data represent average of three independent experiments ± SD. Standard errors are shown as bars above columns. * denotes significant difference at *p* < 0.05, and ** denotes significant difference at *p* < 0.01 according to the Student’s *t*-test.

PEG (polyethylene glycol) and NaCl stress can cause similar cell damage, leading to osmotic stress [29]. Plants adapt to and respond to drought and salt stress by inducing the expression of a series of genes. ABA (abscisic acid) is an important plant hormone that regulates the expression of stress response genes in plants [30]. In order to explore the response of the *TaOSCA* genes to these three abiotic stresses, we analyzed the expression profiles of 15 representative members treated with exogenous PEG, NaCl, or ABA for 4 h, 12 h, and 24 h, respectively. All 15 test genes were all up-regulated at 12 and 24 h after PEG treatment, but some members were significantly down-regulated at 4 h (Figure 6). *TaOSCA21*/*-39* showed the largest increases at 24 h, up 12 times and 15 times, respectively (Figure 6).

Following NaCl treatment, most of the members were down-regulated at 4 h but with no effect at 12 and 24 h, including *TaOSCA2*/-*6*/-*12*/-*15*/-*19*//-*21*/-*27*/-*32*/-*33*/-*34*/-*36*/-*37*/-*40* (Figure 6). *TaOSCA39* was down-regulated at 4 h, unchanged at 12 h, and doubled at 24 h. *TaOSCA1* was significantly inhibited at 4 and 12 h, but unchanged at 24 h (Figure 6).

Following ABA treatment, *TaOSCA21* was up-regulated by five times at 24 h, *TaOSCA39* was up-regulated by five times at 12 h and 24 h, and *TaOSCA40* was up-regulated by two times at 12 h. Except for *TaOSCA21*/-*39*, the other members were all down-regulated at 4 h (Figure 6).

## 3. Discussion

Water is essential for the growth and development of plants. The lack of environmental water triggers an osmotic stress signal cascade, which induces short-term cellular responses to reduce water loss, and long-term responses to reshape the transcription network and physiological development processes [31,32]. The perception of biotic and abiotic stresses often leads to the closure of plant stomata [1,22]. The rapid influx of Ca^2+^ through the plasma membrane plays an important role in this reaction [23,24]. Current research suggests that OSCA family members act as osmotic sensors to mediate the increase in intracellular Ca^2+^ concentration induced by hyperosmolality and control the closure of stomata in the process of immune signal transduction [15,25]. In view of the importance of the functions of OSCA family members, they have been systematically identified in several species, including *A. thaliana*, rice, maize, and *Vigna radiata* (L.) [15,16,17,18] but not so far in wheat. In this study, 42 OSCA members were identified using *T. aestivum* genomic data, and the expression patterns of 15 members in different tissues and under three abiotic stress treatments were analyzed. The results of the study lay the foundation for further research on the function of *OSCA* genes and the cultivation of new *T. aestivum* varieties.

The number of OSCAs in the *T. aestivum* genome is more than that of *A. thaliana*, rice, maize, and *Vigna radiate* [15,16,17,18], perhaps in part because the *T. aestivum* genome is larger. Phylogenetic analysis showed that TaOSCAs fall into four clades (Figure 1), consistent with the topology of the phylogenetic tree of *A. thaliana* OSCA members [15]. The TaOSCAs in clades I, II, and III are relatively highly conserved, and the composition patterns of the conserved motifs in the three branches are similar (Figure 2A,B). The TaOSCAs in clade IV have only four conserved motifs, much less than other TaOSCAs, and the gene structure is quite different from the members of the other clades (Figure 2). Interestingly, the three members of this clade have one less pfam13967 domain than other members in terms of domain composition (Figure 3), suggesting that these three members may have unique functions or special functional mechanisms.

The cis-acting elements predicted in the *TaOSCA* promoter region included environmental stress-related elements, hormone-responsive elements, development-related elements, and light-responsive elements (Figure 4A). Environmental stress-related elements include STRE/DRE1/ARE/TC-rich repeats and so on (Figure 4B). STRE elements are activated by heat shock, osmotic stress, low pH, nutrient starvation, etc. DRE1 is induced by drought and osmotic stress, ARE is induced by anaerobiosis, and TC-rich repeats play a role in response to defense [33]. This suggests that *TaOSCA* genes are involved in the signal transduction network of various stresses in *T. aestivum*. Consistent with this, the expression of multiple genes was significantly up-regulated under PEG/ABA/NaCl treatment. For example, *TaOSCA21*/-*39* were up-regulated by about 10 times when treated with PEG for 24 h, while these two genes were up-regulated by about five times when treated with ABA for 24 h (Figure 6), indicating that these two members can respond simultaneously to PEG and ABA.

Studies on the members of the OSCA family in *A. thaliana* show that different members participate in response to different stress events [15,21,25]. For example, OSCA3.1 has been reported to be an early response protein to dehydration stress [21]. OSCA1.1 is a hyperosmolality sensor, and OSCA1.3 controls the influx of Ca^2+^ in the process of immune signal transduction [15,25]. A similar differentiation in function was observed in our gene expression results. *TaOSCA21*/-*39* were significantly induced by PEG and ABA but were inhibited by NaCl treatment for 4 h (Figure 6). Following NaCl treatment for 24 h, there was no significant effect on the expression of *TaOSCA21*, but *TaOSCA39* was up-regulated by two times (Figure 6). *TaOSCA40* had no obvious response to NaCl, but its expression was up-regulated by two times when treated with ABA for 12 h (Figure 6). The number of development-related elements is less than the other three types of elements (Figure 4A), suggesting that the main function of *TaOSCAs* is to mediate environmental stress signal transduction.

## 4. Materials and Methods

### 4.1. TaOSCA Gene Identification

The protein sequences of 15 *A. thaliana OSCA* genes were downloaded from TAIR. The newly released reference genome of bread wheat (*Triticum aestivum*) used in this study was downloaded from the Ensemble Plants database (http://plants.ensembl.org/Triticum_aestivum/Info/Index, accessed on 29 December 2021). TaOSCA was identified through two rounds of BLASTP referring to Wu et al. and Chen et al. [34,35]. Firstly, all *A. thaliana* OSCA sequences were used to search for possible TaOSCA sequences through TBtools [36]. Then, NCBI’s Batch CD-Search function was used to confirm whether the candidate TaOSCA had the conserved DUF221 domain (pfam accession number: 02714) and other typical domains. Those that did not meet the candidate conditions were eliminated. The predicted CDS length, PI, and molecular weight of the TaOSCA proteins were determined by ExPASy [37].

### 4.2. Phylogenetic Analysis

AtOSCA and TaOSCA full-length protein sequences were used to construct phylogenetic trees in MEGAX using neighbor-joining (NJ) method with 1000 bootstrap replicates [38].

### 4.3. Conserved Motifs, Gene Structure, and Functional Domain Analysis

Conserved motifs were analyzed with the MEME program, and the maximum number of predicted motifs was set to 10 [39]. The gene structure was analyzed using the *T. aestivum* genome annotation files (downloaded from the Ensemble Plants database) and visualized with TBtools. The NCBI Batch CD-Search function was used to analyze and visualize the functional domains.

### 4.4. Promoter Cis-Acting Element Prediction

In order to study the cis-elements in the promoter region of the *TaOSCA* gene, the 2 kb genomic sequence upstream of the start codon (ATG) of each gene was downloaded from the Ensemble Plants database. The putative cis-regulatory elements in the promoter sequence were analyzed by PlantCARE [33].

### 4.5. Plant Materials and Abiotic Stress Treatments

For quantitative PCR expression analysis, *T. aestivum* seedlings (YangMai 158, shared by Professor Jian Yang from Ningbo University) were raised in potting compost under the following controlled conditions: temperature 23 ± 1 °C; 16 h day/8 h night; and relative humidity 60%. Seedlings at the three-leaf stage were treated with 20% (*w/v*) PEG-6000, NaCl (100 mM), ABA (100 μM), or 0.1% Triton X-100 (control) [17,40]. After the stress treatment, leaves were collected at different time points, quickly frozen in liquid nitrogen, and stored at −80 °C. RNA was then extracted to analyze the expression patterns of different *OSCA* genes.

### 4.6. RNA Isolation and Expression Analysis of TaOSCA Genes

Total RNA was extracted by the TRIZOL method, and 1 μg total RNA was used for reverse transcription using Toyobo cDNA First Strand Synthesis Kit. Subsequently, RT-qPCR was performed on the Roche LightCycler^®^ 480 Real-Time PCR instrument with Toyobo Premix Kit. Three independent biological replicates and three technical replicates were adopted. The *T. aestivum cell division cycle* (*CDC*) gene (accession number XM_020313450) was used as the internal reference gene, and the relative expression of the gene was calculated by the 2^−ΔΔC(t)^ method [41]. All primers are listed in the Supplementary Table S4.

## 5. Conclusions

In this study, we identified 42 OSCA members of *T. aestivum* and systematically analyzed their phylogeny, gene structure, conserved domains, cis-acting elements, tissue-specific expression, and transcriptional response to different abiotic stresses. The results show that all 15 *TaOSCA* members tested responded to PEG treatment, while *TaOSCA12*/-*39* responded simultaneously to PEG and ABA, indicating that *T. aestivum OSCA* genes play important roles in regulating the response of plants to various abiotic stresses. The whole genome identification and characterization of the members of the *OSCA* family in *T. aestivum* is an important starting point for further in-depth study of the function of the gene family and lays the foundation for the breeding and genetic improvement of *T. aestivum*.

## Figures and Tables

**Table 1:** Detailed information of the 42 predicted OSCA proteins in *T. aestivum*.

Gene Symbol	Gene Locus	Chromosome Position	CDS (bp)	Protein Length (aa)	Theoretical PI	Molecular Weight (kDa)
TaOSCA1	TraesCS1A02G029300.1	1A:13762566:13768572	2295	764	8.94	87.7
TaOSCA2	TraesCS1A02G034100.1	1A:16913591:16919361	2301	766	9.06	87.9
TaOSCA3	TraesCS1A02G196800.1	1A:354950050:354955890	2412	803	9.15	93.4
TaOSCA4	TraesCS1A02G247300.1	1A:438692887:438699043	2304	767	7.33	87.6
TaOSCA5	TraesCS1B02G036500.1	1B:17379054:17386839	2295	764	8.81	87.8
TaOSCA6	TraesCS1B02G211400.1	1B:384496418:384502005	2406	801	9.12	93.1
TaOSCA7	TraesCS1B02G257900.1	1B:453873827:453880440	2304	767	7.73	87.6
TaOSCA8	TraesCS1D02G029500.1	1D:11616634:11624592	2295	764	8.87	87.8
TaOSCA9	TraesCS1D02G035600.1	1D:16402928:16408856	2301	766	9.09	87.9
TaOSCA10	TraesCS1D02G200300.1	1D:282959933:282965842	2409	802	9.16	93.2
TaOSCA11	TraesCS1D02G246500.1	1D:339106416:339112794	2301	766	7.73	87.5
TaOSCA12	TraesCS2A02G252100.1	2A:383596822:383601415	2097	698	9.17	78.1
TaOSCA13	TraesCS2A02G262100.1	2A:413328225:413332576	2259	752	8.83	85.1
TaOSCA14	TraesCS2B02G271900.1	2B:373476491:373481152	2178	725	9.29	81.2
TaOSCA15	TraesCS2B02G280100.1	2B:386306102:386311122	2307	768	6.59	87.4
TaOSCA16	TraesCS2D02G252800.1	2D:304963347:304967946	2016	671	9.27	75.4
TaOSCA17	TraesCS2D02G261900.1	2D:318328838:318333878	2307	768	6.72	87.4
TaOSCA18	TraesCS3A02G019900.1	3A:11891585:11895285	2178	725	9.42	81.7
TaOSCA19	TraesCS3B02G231400.1	3B:349934463:349949455	2214	737	8.58	84.3
TaOSCA20	TraesCS3B02G541500.1	3B:779622377:779627065	2130	709	8.97	80.0
TaOSCA21	TraesCS3D02G026700.1	3D:9094139:9098190	1947	648	9.51	72.5
TaOSCA22	TraesCS3D02G201300.1	3D:238617165:238630499	2214	737	8.58	84.2
TaOSCA23	TraesCS3D02G485000.1	3D:581939165:581943412	2130	709	9.01	80.1
TaOSCA24	TraesCS4A02G257600.1	4A:570460851:570465209	2238	745	8.89	85.7
TaOSCA25	TraesCS4A02G291700.1	4A:594680010:594686528	2301	766	9.33	87.0
TaOSCA26	TraesCS4B02G022300.1	4B:16056589:16063157	2301	766	9.44	86.8
TaOSCA27	TraesCS4B02G023400.1	4B:17037475:17042314	1410	469	9.77	53.7
TaOSCA28	TraesCS4B02G056900.1	4B:46623552:46628077	2238	745	8.84	85.7
TaOSCA29	TraesCS4B02G335800.1	4B:627519072:627524699	2385	794	7.2	89.6
TaOSCA30	TraesCS4D02G020100.1	4D:8653451:8660027	2229	742	9.13	84.3
TaOSCA31	TraesCS4D02G057200.1	4D:32238120:32242237	2238	745	8.84	85.7
TaOSCA32	TraesCS4D02G331300.1	4D:489186164:489190977	2385	794	7.55	89.5
TaOSCA33	TraesCS5A02G012500.1	5A:8233156:8239545	2415	804	9.07	90.7
TaOSCA34	TraesCS5A02G012600.1	5A:8240521:8246498	2043	680	8.04	77.2
TaOSCA35	TraesCS5A02G071400.1	5A:80159622:80167881	2097	698	8.71	78.9
TaOSCA36	TraesCS5A02G505500.1	5A:670818817:670823201	2385	794	7.55	89.6
TaOSCA37	TraesCS5B02G010700.1	5B:10438285:10442125	2286	761	8.96	86.3
TaOSCA38	TraesCS5B02G010800.1	5B:10442174:10450187	2319	772	9.07	87.4
TaOSCA39	TraesCS5B02G077400.1	5B:93218161:93227257	2094	697	8.62	79.1
TaOSCA40	TraesCS5D02G018000.1	5D:10520893:10527152	2334	777	9.08	88.1
TaOSCA41	TraesCS5D02G018100.1	5D:10537542:10542334	2316	771	8.61	87.4
TaOSCA42	TraesCS5D02G083600.1	5D:87182360:87193201	2097	698	8.79	79.0

## Data Availability

Not applicable.

## Supplementary Materials

The following supporting information can be downloaded at: https://www.mdpi.com/article/10.3390/ijms23010469/s1.

## References

[1] Melotto M., Zhang L., Oblessuc P.R., He S.Y. (2017). Stomatal defense a decade later. Plant Physiol..

[2] Chen X., Ding Y., Yang Y., Song C., Wang B., Yang S., Guo Y., Gong Z. (2021). Protein kinases in plant responses to drought, salt, and cold stress. J. Integr. Plant Biol..

[3] Bartels D., Sunkar R. (2005). Drought and salt tolerance in plants. CRC Crit. Rev. Plant Sci..

[4] McAinsh M.R., Pittman J.K. (2009). Shaping the calcium signature. New Phytol..

[5] Kaur H., Sirhindi G., Bhardwaj R., Alyemeni M.N., Siddique K.H., Ahmad P. (2018). 28-homobrassinolide regulates antioxidant enzyme activities and gene expression in response to salt and temperature-induced oxidative stress in *Brassica juncea*. Sci. Rep..

[6] Berridge M.J., Lipp P., Bootman M.D. (2000). The versatility and universality of calcium signalling. Nat. Rev. Mol. Cell Biol..

[7] Knight H., Trewavas A.J., Knight M.R. (1997). Calcium signalling in *Arabidopsis thaliana* responding to drought and salinity. Plant J..

[8] Dodd A.N., Kudla J., Sanders D. (2010). The language of calcium signaling. Annu. Rev. Plant Biol..

[9] Kung C. (2005). A possible unifying principle for mechanosensation. Nature.

[10] Arnadottir J., Chalfie M. (2010). Eukaryotic mechanosensitive channels. Annu. Rev. Biophys..

[11] Zhu J.K. (2002). Salt and drought stress signal transduction in plants. Annu. Rev. Plant Biol..

[12] Monshausen G.B., Gilroy S. (2009). Feeling green: Mechanosensing in plants. Trends Cell Biol..

[13] Swarbreck S.M., Colaco R., Davies J.M. (2013). Plant calcium-permeable channels. Plant Physiol..

[14] Hou C., Tian W., Kleist T., He K., Garcia V., Bai F., Hao Y., Luan S., Li L. (2014). DUF221 proteins are a family of osmosensitive calcium-permeable cation channels conserved across eukaryotes. Cell Res..

[15] Yuan F., Yang H., Xue Y., Kong D., Ye R., Li C., Zhang J., Theprungsirikul L., Shrift T., Krichilsky B.L. (2014). OSCA1 mediates osmotic-stress-evoked Ca^2+^ increases vital for osmosensing in Arabidopsis. Nature.

[16] Li Y., Yuan F., Wen Z., Li Y., Wang F., Zhu T., Zhuo W., Jin X., Wang Y., Zhao H. (2015). Genome-wide survey and expression analysis of the OSCA gene family in rice. BMC Plant Biol..

[17] Yin L., Zhang M., Wu R., Chen X., Liu F., Xing B. (2021). Genome-wide analysis of OSCA gene family members in *Vigna radiata* and their involvement in the osmotic response. BMC Plant Biol..

[18] Ding S., Feng X., Du H., Wang H. (2019). Genome-wide analysis of maize OSCA family members and their involvement in drought stress. PeerJ.

[19] Schroeder B.C., Cheng T., Jan Y.N., Jan L.Y. (2008). Expression cloning of TMEM16A as a calcium-activated chloride channel subunit. Cell.

[20] Liu X., Wang J., Sun L. (2018). Structure of the hyperosmolality-gated calcium-permeable channel OSCA1.2. Nat. Commun..

[21] Kiyosue T., Yamaguchi-Shinozaki K., Shinozaki K. (1994). Cloning of cDNAs for genes that are early-responsive to dehydration stress (ERDs) in *Arabidopsis thaliana* L.: Identification of three ERDs as HSP cognate genes. Plant Mol. Biol..

[22] Sussmilch F.C., Schultz J., Hedrich R., Roelfsema M.R.G. (2019). Acquiring control: The evolution of stomatal signalling pathways. Trends Plant. Sci..

[23] Hedrich R. (2012). Ion channels in plants. Physiol. Rev..

[24] Jezek M., Blatt M.R. (2017). The membrane transport system of the guard cell and its integration for stomatal dynamics. Plant Physiol..

[25] Thor K., Jiang S., Michard E., George J., Scherzer S., Huang S., Dindas J., Derbyshire P., Leitão N., DeFalco T.A. (2020). The calcium-permeable channel OSCA1.3 regulates plant stomatal immunity. Nature.

[26] Pfeifer M., Kugler K.G., Sandve S.R., Zhan B., Rudi H., Hvidsten T.R., Mayer K.F.X., Olsen O.-A., International Wheat Genome Sequencing Consortium (2014). Genome interplay in the grain transcriptome of hexaploid bread wheat. Science.

[27] Wadskog I., Forsmark A., Rossi G., Konopka C., Öyen M., Goksör M., Ronne H., Brennwald P., Adler L. (2006). The yeast tumor suppressor homologue Sro7p is required for targeting of the sodium pumping ATPase to the cell surface. Mol. Biol. Cell.

[28] Zhu J., Zhang B., Smith E.N., Drees B., Brem R.B., Kruglyak L., Bumgarner R.E., Schadt E.E. (2008). Integrating large-scale functional genomic data to dissect the complexity of yeast regulatory networks. Nat. Genet..

[29] Wang W., Vinocur B., Altman A. (2003). Plant responses to drought, salinity and extreme temperatures: Towards genetic engineering for stress tolerance. Planta.

[30] Chen K., Li G.J., Bressan R.A., Song C.P., Zhu J.K., Zhao Y. (2020). Abscisic acid dynamics, signaling, and functions in plants. J. Integr. Plant Biol..

[31] Cutler S.R., Rodriguez P.L., Finkelstein R.R., Abrams S.R. (2010). Abscisic acid: Emergence of a core signaling network. Annu. Rev. Plant Biol..

[32] Kim T.H., Böhmer M., Hu H., Nishimura N., Schroeder J.I. (2010). Guard cell signal transduction network: Advances in understanding abscisic acid, CO_2_, and Ca^2+^ signaling. Annu. Rev. Plant Biol..

[33] Lescot M., Déhais P., Thijs G., Marchal K., Moreau Y., Van de Peer Y., Rouze P., Rombauts S. (2002). PlantCARE, a database of plant cis-acting regulatory elements and a portal to tools for in silico analysis of promoter sequences. Nucleic Acids Res..

[34] Wu X., Lai Y., Lv L., Ji M., Han K., Yan D., Lu Y., Peng J., Rao S., Yan F. (2020). Fasciclin-like arabinogalactan gene family in *Nicotiana benthamiana*: Genome-wide identification, classification and expression in response to pathogens. BMC Plant Biol..

[35] Chen X., Wu X., Qiu S., Zheng H., Lu Y., Peng J., Wu G., Chen J., Rao S., Yan F. (2021). Genome-wide identification and expression profiling of the BZR transcription factor gene family in *Nicotiana benthamiana*. Int. J. Mol. Sci..

[36] Chen C., Chen H., Zhang Y., Thomas H.R., Frank M.H., He Y., Xia R. (2020). TBtools: An integrative toolkit developed for interactive analyses of big biological data. Mol. Plant.

[37] Wilkins M.R., Gasteiger E., Bairoch A., Sanchez J.-C., Williams K.L., Appel R.D., Hochstrasser D.F. (1999). Protein identification and analysis tools in the ExPASy server. Methods Mol. Biol..

[38] Kumar S., Stecher G., Li M., Knyaz C., Tamura K. (2018). MEGA X: Molecular evolutionary genetics analysis across computing platforms. Mol. Biol. Evol..

[39] Bailey T.L., Boden M., Buske F.A., Frith M., Grant C.E., Clementi L., Ren J., Li W.W., Noble W.S. (2009). MEME Suite: Tools for motif discovery and searching. Nucleic Acids Res..

[40] He L., Chen X., Xu M., Liu T., Zhang T., Li J., Yang J., Chen J., Zhong K. (2021). Genome-wide identification and characterization of the cystatin gene family in bread wheat (*Triticum aestivum* L.). Int. J. Mol. Sci..

[41] Livak K.J., Schmittgen T.D. (2001). Analysis of relative gene expression data using real-time quantitative PCR and the 2^−ΔΔCT^ Method. Methods.

